# Isolation and Characterization of a Novel Gammaherpesvirus from a Microbat Cell Line

**DOI:** 10.1128/mSphere.00070-15

**Published:** 2016-02-17

**Authors:** Reed S. Shabman, Susmita Shrivastava, Tshidi Tsibane, Oliver Attie, Anitha Jayaprakash, Chad E. Mire, Kari E. Dilley, Vinita Puri, Timothy B. Stockwell, Thomas W. Geisbert, Ravi Sachidanandam, Christopher F. Basler

**Affiliations:** a Virology Group, J. Craig Venter Institute, Rockville, Maryland, USA; b Department of Microbiology, Icahn School of Medicine at Mount Sinai, New York, New York, USA; c Department of Genetics and Genomic Sciences, Icahn School of Medicine at Mount Sinai, New York, New York, USA; d Department of Microbiology and Immunology, University of Texas Medical Branch, Galveston, Texas, USA; e Department of Oncological Sciences, Icahn School of Medicine at Mount Sinai, New York, New York, USA; UNC-Chapel Hill

**Keywords:** bats, genomics, herpesviruses, next-generation sequencing, transcriptomics, virus discovery

## Abstract

While employing deep sequencing and *de novo* assembly to characterize the mRNA transcript profile of a cell line derived from the microbat *Myotis velifer incautus*, we serendipitously identified mRNAs encoding proteins with a high level of identity to herpesviruses. A majority were closely related to proteins of equine herpesvirus 2 (EHV-2), a horse gammaherpesvirus. We demonstrated by electron microscopy the presence of herpesvirus-like particles in the microbat cells. Passage of supernatants from microbat cells to Vero cells resulted in syncytium formation, and expression of viral genes and amplification of viral DNA were demonstrated by quantitative PCR. Susceptibility of human cell lines to productive infection was also demonstrated. Next-generation sequencing and *de novo* assembly of the viral genome from supernatants from Vero cells yielded a single contig of approximately 130 kb with at least 77 open reading frames (ORFs), predicted microRNAs (miRNAs), and a gammaherpesvirus genomic organization. Phylogenic analysis of the envelope glycoprotein (gB) and DNA polymerase (POLD1) revealed similarity to multiple gammaherpesviruses, including those from as-yet-uncultured viruses of the *Rhadinovirus* genus that were obtained by deep sequencing of bat tissues. Moreover, the assembled genome revealed ORFs that share little or no homology to known ORFs in EHV-2 but are similar to accessory proteins of other gammaherpesviruses. Some also have striking homology to predicted *Myotis* bat proteins. Cumulatively, this study provides the first isolation and characterization of a replication-competent bat gammaherpesvirus.

**IMPORTANCE** Bats are of significant interest as reservoirs for zoonotic viral pathogens; however, tools to dissect bat-virus interactions are limited in availability. This study serendipitously identified, in an established bat cell line, a fully replication-competent gammaherpesvirus; determined the complete genome sequence of the virus; and generated a viral transcript map. This virus can replicate in select human and nonhuman primate cell lines. However, analyses of viral sequences support a bat origin for this virus; we therefore refer to the virus as bat gammaherpesvirus 8 (BGHV8). The viral genome contains unique open reading frames that likely encode modulators of bat innate and adaptive immune signaling pathways and expresses viral miRNAs. The virus and its gene products should provide a unique tool to dissect both bat and gammaherpesvirus biology.

## Abstract (precis)

Bats are of significant interest as reservoirs for zoonotic viral pathogens; however, tools to dissect bat-virus interactions are limited in availability. This study serendipitously identified, in an established bat cell line, a fully replication-competent gammaherpesvirus; determined the complete genome sequence of the virus; and generated a viral transcript map. This virus can replicate in select human and nonhuman primate cell lines. However, analyses of viral sequences support a bat origin for this virus; we therefore refer to the virus as bat gammaherpesvirus 8 (BGHV8). The viral genome contains unique open reading frames that likely encode modulators of bat innate and adaptive immune signaling pathways and expresses viral miRNAs. The virus and its gene products should provide a unique tool to dissect both bat and gammaherpesvirus biology.

## INTRODUCTION

A majority of all emerging infectious diseases (EIDs) in humans arise from zoonotic pathogens (1). Zoonotic viruses are harbored across animal species, but bats have drawn particular interest as potential sources of such pathogens. Bats comprise 20% of all known mammal species (2) and belong to the order Chiroptera, which is divided into two suborders, Megachiroptera (megabats), containing a single family, Pteropodidae, with 42 genera, and Microchiroptera (microbats), containing 16 families with 135 genera. Frequent associations of bat viruses with zoonotic viral diseases of humans and the fact that bats are described as hosts for a large variety of both DNA and RNA viruses suggest an unusual capacity to harbor viruses (reviewed in reference 3). However, a complete understanding of why bats can accommodate viruses that are often highly lethal in humans requires a more detailed characterization of bat-virus interactions than is currently available. Challenges to a more complete understanding of bat-virus interactions include the limited number of reagents such as readily available cell lines from diverse bat species, the absence of fully annotated genomes, limited reagents to characterize bat responses to infections, and a limited number of well-characterized viruses that naturally infect bats.

Rapidly evolving next-generation sequencing (NGS) technologies can facilitate bat-virus interaction studies both by identifying viruses that infect bats and by allowing a characterization of host responses to virus infection. For example, shotgun metagenomic studies have identified viral, bacterial, and eukaryotic sequences present in both environmental and animal samples (4, 5). In this approach, sequencing is obtained directly from the sample source, and read data are then processed to identify sequence homology to known organisms through metagenomics analysis software tools (6–8). A benefit of the shotgun metagenomics approach is that viruses unable to be propagated are readily identified. Alternative virus discovery approaches utilize degenerate primers specific for a virus family and/or random primers for signal amplification and subsequent NGS library construction. A recent study highlighting the power of the latter approaches used conserved primers across RNA and DNA virus families to sample the viral diversity in the *Pteropus giganteus* bat (9). Sequencing of over 12,000 amplicons identified both known and novel virus sequences across nine virus families (9). Transcriptomics and metatranscriptomics, analyzed historically through microarray and more recently by NGS, represent an approach that can both identify viral sequences in a given sample and characterize host responses to infections (reviewed in reference 10). Generally, mRNA is isolated from a sample, and postsequencing analysis allows for the identification of both host and viral mRNAs. This sequence-independent method allows for the identification of actively transcribed viral and host messages. Moreover, transcriptomic analyses by NGS from large DNA viruses can identify temporal gene expression patterns and transcriptional start and stop sites (11, 12) and can also define splice variants and novel protein coding transcripts (13). A limitation of these approaches is that while they can rapidly identify genomic viral sequences, they often uncover only partial sequences (hundreds of bases of sequence or less) and rarely identify replication-competent virus. However, upon identification of viral sequences, approaches such as random priming and conserved primer approaches can be used to determine full-length viral genomic sequences, as illustrated by the discovery of severe acute respiratory syndrome (SARS) virus in passaged cultures obtained from infected individuals (14, 15).

Initiated with the goal of characterizing the response of a microbat cell line to virus infection, the present study employed deep sequencing of bat cell mRNAs and serendipitously identified the presence of transcripts corresponding to a novel gammaherpesvirus. Follow-up studies demonstrated that the bat cell virus is released in a replication-competent form and can productively replicate in several nonhuman primate and human cell lines. Phylogenetic studies on this virus sequence suggest a bat origin for the new gammaherpesvirus. Although previous reports have described other gammaherpesvirus sequences from bats (16–18), as well as a replication-competent bat betaherpesvirus (19), the present study describes the first isolation of what is likely a bat gammaherpesvirus. The transcriptome data from the bat cell line also provide a transcript map for the novel gammaherpesvirus. Of note, novel open reading frames (ORFs) that likely function to regulate the bat immune responses appear to be expressed. Taken together, the gammaherpesvirus and microbat cell line provide a platform that will enable a greater understanding of both bat cell biology and innate immune regulatory mechanisms.

## RESULTS

### Identification of gammaherpesvirus transcripts in a microbat cell line.

We obtained a *Myotis velifer incautus* cell line that was originally derived from an interscapular tumor (MVI-it) and that was generated at the Naval Biosciences Laboratory and deposited at the American Type Culture Collection (ATCC; catalogue no. CRL-6012). The growth properties of this cell line changed over several weeks in culture, from doubling approximately once a week to rapidly proliferating, doubling every 2 to 3 days. We profiled the transcriptome of the cell line after it had begun rapid growth. Total RNA was isolated, and mRNA was purified and subjected to transcriptome sequencing (RNA-seq) followed by an initial *de novo* assembly that generated approximately 5,000 contigs. Unexpectedly, within this list of contigs 23 that exhibited homology to herpesvirus mRNAs were identified, suggesting the possibility that the cell line might harbor a replicating herpesvirus (Table 1). The viral transcripts detected included those for structural proteins, such as the major capsid protein, tegument protein, and glycoprotein B (gB), as well as proteins involved in genome replication, such as DNA polymerase and thymidine kinase, suggesting ongoing replication. A majority of the mRNAs potentially encoding viral proteins had a high level of identity (approximately 42 to 75% amino acid identity) to proteins of equine herpesvirus 2 (EHV-2), a horse gammaherpesvirus. Since each of these viral sequences showed substantial differences from any sequences deposited in NCBI, we refer to the virus as bat gammaherpesvirus 8 (BGHV8) based on suggested International Committee on the Taxonomy of Viruses (ICTV) nomenclature (20) and described bat gammaherpesvirus sequences (3).

**TABLE 1:** *De novo* assembly finds transcripts from a novel gammaherpesvirus

Protein type and assembly gene identifier^a^	Contig length^b^	Homologous gammaherpesvirus (protein product)^c^
Structural proteins		
BatA76	441	EHV-2 (glycoprotein B)
BatA222	354	*Myotis ricketti* herpesvirus 2 (glycoprotein B)
BatA401	420	EHV-2 (glycoprotein H)
BatA1312	168	EHV-2 (ORF23)
BatA64	333	EHV-2 (ORF33)
BatA215	1,140	EHV-2 (major capsid protein)
BatA305	1,020	EHV-2 (tegument protein)
BatA21	1,174	EHV-2 (tegument protein)
BatA4	300	EHV-2 (capsid triplex subunit 2)
BatA100	576	EHV-2 (proteinase/capsid scaffold protein)
BatA985	419	EHV-2 (tegument)
BatA166	300	EHV-2 (ORF69)
Nonstructural proteins		
BatA168	140	EHV-2 (ORF37)
BatA892	299	EHV-2 (ORF50 and partial ORF49)
BatA242	354	EHV-2 (ribonucleotide reductase large subunit)
BatA232	310	EHV-2 (ssDNA binding protein)
BatA42	480	EHV-2 (ssDNA binding protein)
BatA295	572	EHV-2 (thymidine kinase)
BatA548	600	EHV-2 (DNA polymerase)
BatA1061	374	EHV-2 (DNA helicase-primase complex component)
BatA162	79	EHV-2 (ORF45)
BatA85	110	EHV-2 (ORF52)
BatA260	289	EHV-2 (partial ORF31)

a A total of 5,818 contigs were generated from *de novo*-assembled RNA-seq data from the CRL-6012 cell line. Contigs were labeled BatA1 to BatA5818.

b The contig length represents the number of amino acids present following translation of each sequence.

c Translated sequences were subjected to a BLAST search, and contigs with high similarity to herpesvirus structural and nonstructural proteins are displayed.

### Multiple primate cell lines are permissive for BGHV8 transcription and replication.

**FIG 1:**
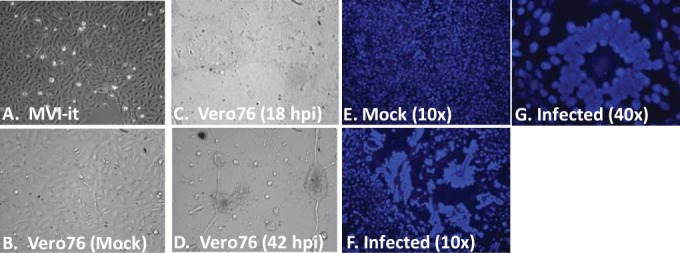
Supernatant transfer from the MVI-it bat cell line to Vero cells results in syncytia and CPE. (A) MVI-it cells growing in culture harboring the novel gammaherpesvirus. (B) Phase-contrast image of Vero cells prior to MVI-it supernatant transfer. (C) Phase-contrast image of Vero cells 18 h after MVI-it supernatant transfer. (D) Phase-contrast image of Vero cells 42 h after supernatant transfer. (E) Hoechst-stained Vero cells from panel B display nuclei prior to MVI-it supernatant transfer. (F and G) Hoechst-stained Vero cells 18 h after supernatant transfer display evidence of syncytia.

**FIG 2:**
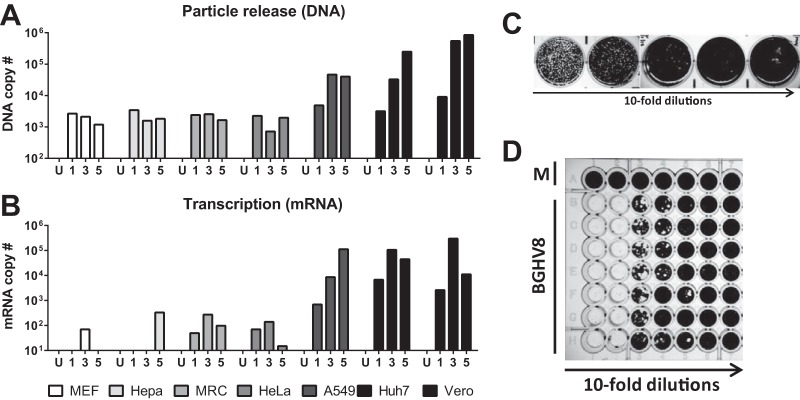
Quantitation of bat gammaherpesvirus 8-infected cells and release in supernatant from multiple cell lines. (A) Quantitative PCR (qPCR) to measure the amount of viral DNA released into the supernatant of seven representative cell lines at days 1, 3, and 5 postinfection. (B) Similar to panel A, but mRNA from infected cells was analyzed by reverse transcription followed by qPCR. (C) Representative plaque assay of BGHV8 on Vero cells. (D) Representative 50% tissue culture infectious dose (TCID_50_) data from BGHV8 on Vero cells.

In an initial attempt to propagate BGHV8, we transferred supernatant from the MVI-it cell line to Vero cells. Within 18 h after supernatant transfer, cytopathic effect (CPE) was observed in the culture (Fig. 1A to D). Moreover, syncytia were observed upon nucleus staining following infection (Fig. 1E to G), a previously described feature of herpesvirus infection *in vitro*, including infection by equine gammaherpesvirus 2 (21). To confirm that the CPE was due to the BGHV8 identified by deep sequencing, we designed quantitative PCR (qPCR) primers based on the mRNA contigs from our RNA-seq data set (see Materials and Methods). To detect viral replication and the release of progeny virions, we isolated DNA from supernatants of cells infected with BGHV8 and measured DNA copy numbers by qPCR at 1, 3, and 5 days postinfection (Fig. 2A). In parallel, total RNA from each cell line was harvested and oligo(dT) primers were used to reverse transcribe mRNA from both mock- and BGHV8-infected cell lines (Fig. 2B). For both samples, signal was measured with primer pairs specific for a BGHV8 capsid gene (related to EHV ORF25). Consistent with previous CPE results, BGHV8 was able to both transcribe mRNA and generate DNA copies in select cell lines (Fig. 2A and B). Specifically, we detected BGHV8 genome amplification and mRNA expression in Vero, A549, and Huh7 cells, cell lines derived from African green monkey kidney, human lung, and human liver, respectively.

### Development of assays to determine titer of BGHV8.

To prove that infectivity of the BGHV8 can be propagated beyond a single passage in cell culture, we passaged the virus multiple times in Vero cells (data not shown). Since the virus was able to be efficiently propagated, we optimized assays to measure infectivity, including a plaque assay (Fig. 2C) and a 50% tissue culture infectious dose (TCID_50_) assay (Fig. 2D) on Vero cells, the most permissive cell line tested. BGHV8 completely destroys Vero monolayers, and the corresponding supernatants produce virus titers between 10^5^ and 10^6^ PFU/TCID_50_ per milliliter of supernatant. Taken together, these data indicate that BGHV8 undergoes lytic replication in Vero cells, allowing infectious virus to be quantified.

### EM confirms the presence of herpesvirus-like particles within the MVI-it culture.

**FIG 3:**
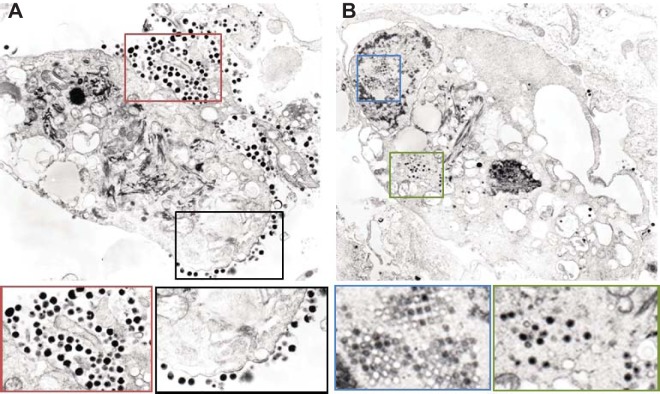
Electron microscopy of the MVI-it cell line identifies viral particles that appear to be BGHV8. (A) Representative EM image from the MVI-it culture. The black and red boxes highlight enlarged regions directly below the image above. Both areas identify budding and fully budded herpesvirus virions. (B) Representative EM image from the MVI-it culture. The blue and green boxes highlight enlarged regions directly below the image above. Both areas highlight intracellular herpesvirus particles, and the blue box captures virions within an inclusion body. Courtesy of Vsevolod Popov and Krystle Agans, reproduced with permission.

Both the MVI-it RNA-seq and the supernatant transfer experiments described above suggested that this cell line actively harbored replicating BGHV8. Therefore, electron microscopy (EM) studies were performed in an attempt to visualize virus particles present in the MVI-it culture (Fig. 3). Viral particles at the plasma membrane, which may represent budding events, were observed (Fig. 3A). Strikingly, arrays of virus capsid-like particles were also visible within the nucleus and enveloped capsid particles were present within the cytoplasm (Fig. 3B). These images are consistent with EM images from gammaherpesvirus-infected cells, including murine gammaherpesvirus infection in mouse NIH 3T3 cells (22), providing additional evidence that the MVI-it cell line persistently harbors replicating BGHV8.

### Construction of full-length annotated genome of BGHV8.

**FIG 4:**
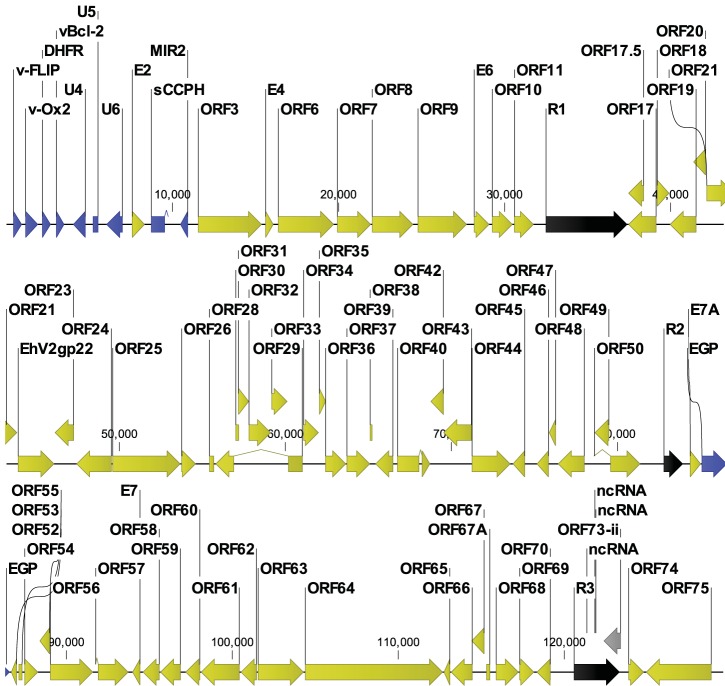
Assembly of the novel bat gammaherpesvirus genome highlights ORFs related to EHV-2 and accessory ORFs related to other gammaherpesviruses. To assemble a single contig of 129,563 bp, contigs assembled with 100-bp reads from Illumina HiSeq were joined with 270-bp reads from Illumina MiSeq data. The genome was annotated using the Viral Genome ORF Reader (VIGOR). Open reading frames are highlighted as follows: yellow, ORFs with homology to EHV-2; blue, ORFs with homology to other herpesvirus proteins; black, repeat regions; gray, putative LANA gene with an internal unresolved repeat region. ncRNA, noncoding RNA.

In order to obtain a complete genomic sequence for BGHV8, supernatant was harvested from infected Vero cells, and DNA was extracted. The DNA was sheared, and a library was generated for sequencing by Illumina HiSeq. *De novo* assembly of the HiSeq reads in CLC Genomics Workbench resulted in several contigs which contained ORFs organized in a manner consistent with a gammaherpesvirus genome. Due to the short length of the HiSeq reads, we were unable to completely assemble a single contig, suggesting that the HiSeq data were not sufficient to assemble through putative repeat regions (a hallmark feature of gammaherpesviruses). In an attempt to close these gaps, we took viral genomic DNA, performed sequence-independent single primer amplification (SISPA), and constructed Illumina sequencing libraries as previously described (23). We next performed MiSeq analysis to obtain sequencing reads approximately 270 bp in length. Using the CLC genome finishing tool, these longer reads were used to join the contigs generated from the HiSeq data into a single sequence of 129,563 bases (Fig. 4). To annotate the assembled BGHV8, we developed gammaherpesvirus-specific annotation software in the Viral Genome ORF Reader (VIGOR) software (24) with relaxed parameters on the percent similarity cutoff, to accommodate the variation in herpesvirus genomes. VIGOR was used to predict genes, perform alignments, ensure the fidelity of open reading frames, and detect any potential sequencing or assembly errors. The annotation was manually verified and edited as required, following the gene and protein naming convention of the *Equid herpesvirus 2* (EHV-2) reference genome (NC_001650.2). Every single gammaherpesvirus-specific gene and the core genes were identified in the annotation (Fig. 4, yellow ORFs) with additional ORFs that did not match EHV-2 ORFs (Fig. 4, blue ORFs). We were not able to fully resolve a repeat region around bp 122000, resulting in a disrupted reading frame for ORF73, which codes for the latency-associated nuclear antigen (LANA; Fig. 4, gray ORF). Strikingly, the organization of the ORFs is consistent with EHV-2 and with other related gammaherpesviruses, and all features are present in the GenBank file KU220026.

### Mapping of tandem repeats and putative miRNAs carried in the BGHV8 genome.

We analyzed the assembled BGHV8 sequence in a tandem repeat finder program (http://tandem.bu.edu/trf/trf.html) that was previously used to identify repeat regions in a mouse gammaherpesvirus genome (25). This analysis highlighted repeat regions, consistent with other gammaherpesvirus genomes (Fig. 4, black arrows). These are in locations similar to those of repeat regions found in other rhadinoviruses, such as Kaposi’s sarcoma-associated herpesvirus/human herpesvirus 8 (KSHV/HHV-8) (26). In addition, a small-RNA capture performed according to established methods (27) to isolate small RNAs identified a large number of putative microRNA (miRNA) sequences within the MVI-it cell line (data not shown). Analysis of these MVI-it small RNAs yielded sequences mapping to three independent regions within the BGHV8 genome between positions 121326 and 121820. These small RNAs are located in a BGHV8 repeat region similar to a location of microRNAs carried by KSHV (26). Therefore, we hypothesize that these sequences may reflect the production of small processed RNAs (such as miRNAs) during BGHV8 infection within the MVI-it cell culture.

### Phylogenic analysis of BGHV8 gB.

**FIG 5:**
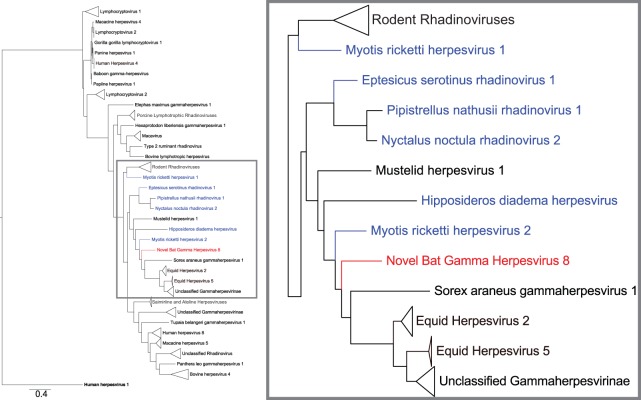
gB protein phylogenic tree highlights similarities with other bat herpesviruses and EHV-2. The tree displays multiple gammaherpesvirus protein sequences, and human herpesvirus 1 represents the outgroup. BGHV8 gB is an 839-amino-acid protein. An alignment was generated for a 263-amino-acid region of gB from positions 498 to 760 of BGHV8. All sequences were trimmed to include only the corresponding region, and an alignment was derived with an LG substitution model and 100 bootstraps with a scale of 0.4. The inset on the right side displays an enlarged image highlighted corresponding to the gray box in the left image to display the sequences most closely related to BGHV8. Red denotes BGHV8, and blue labels denote gB from viruses obtained from bat species. The most closely related sequence from this analysis comes from a gB sequence of *Myotis ricketti* herpesvirus 2 (AFM85235.1). Triangles represent multiple sequences which were collapsed to enhance presentation. To see the tree with no collapsed sequences and corresponding accession numbers, see Fig. S1 in the supplemental material.

To determine the relatedness of BGHV8 to other gammaherpesviruses, a phylogenetic analysis was performed with the gB glycoprotein sequence. gB was chosen in part because, although full sequences of other bat gammaherpesviruses have not been reported, gB sequences have been amplified from bats, suggesting the presence of gammaherpesvirus in these animals. All sequences were trimmed, and the alphaherpesvirus human herpesvirus 1 gB protein served as an outgroup. We then generated a phylogenetic tree for the protein (with the LG substitution model and 100 bootstraps) and rooted it on the outgroup. This analysis confirms that BGHV8 is similar to, but distinct from, other gammaherpesviruses (28), including other apparent bat gammaherpesviruses, and clusters with gB sequences of multiple rhadinovirus sequences (Fig. 5). Strikingly, a majority of these rhadinovirus sequences belong to sequences obtained from microbats, highlighted in blue in Fig. 5. Moreover, the gBs from EHV-2 and EHV-5 also are similar to BGHV8 gB, consistent with the notion that bat and horse species are closely related (29). A more detailed tree without collapsed sequences and accession numbers is provided as Fig. S1 in the supplemental material. Similar results were obtained with a POLD1 phylogenic analysis (data not shown).

10.1128/mSphere.00070-15.1 Figure S1  Similar to Fig. 5, this tree displays each accession number and has no collapsed sequences. The phylogenetic tree was constructed using 100 iterations, and the bootstrap values are displayed at each node. Download Figure S1, EPS file, 1.6 MB. Copyright © 2016 Shabman et al. 2016 Shabman et al. This content is distributed under the terms of the Creative Commons Attribution 4.0 International license.

### Identification and analysis of ORFs that do not share homology with EHV-2 ORFs.

**FIG 6:**
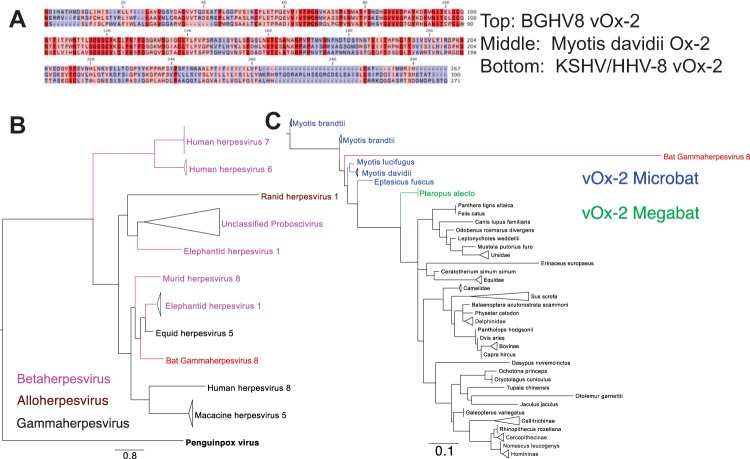
BGHV8 v-OX2 clusters with gammaherpesviruses and *Myotis* sequences. (A) Representative alignment of BGHV8 OX2 with KSHV v-OX2 and *Myotis davidii* OX2. Dark red indicates identical residues, pink indicates conservation between two of the three alignments, and blue indicates less conserved regions. (B) Tree of representative viral OX2 ORFs demonstrates the relationship of BGHV8 v-OX2 to other gammaherpesviruses. Bold black denotes the penguinpox virus v-OX2 outgroup, black indicates gammaherpesvirus v-OX2, and pink denotes betaherpesvirus v-OX2. The red label highlights the location of BGHV8. (C) Tree of representative mammalian OX2 ORFs demonstrates the relatedness of BGHV8 OX2 to those from bat species. Blue labels denote microbat sequences, and green labels denote megabat sequences. The red label highlights the location of BGHV8.

We sought to further characterize the features of the ORFs at the 5′ end of the BGHV8 genome that share little or no homology with the EHV-2. Each ORF highlighted in blue in Fig. 4 was subjected to a BLAST search against both the viral protein database and the mammalian protein database. BLAST results against the viral protein database indicated that seven of the 10 blue ORFs were similar to proteins in other herpesviruses, and therefore, these were assigned a name standard to other gammaherpesvirus accessory genes. These include v-FLIP, E11 (v-OX2), dihydrofolate reductase (DHFR), v-Bcl-2, CCP, and MIR2 ORFs at the far 5′ end of the genome (Fig. 4). An alignment of the BGHV8 v-OX2 aligned with the KSHV v-OX2 (Fig. 6A) highlights conserved regions in red, and a phylogenic analysis illustrates that BGHV8 v-OX2 clusters with v-OX2 proteins of other gammaherpesviruses (Fig. 6B). Phylogenic analysis of the other ORFs confirmed the relatedness of additional BGHV8 ORFs with other gammaherpesvirus ORFs (data not shown). In addition, a single nonhomologous ORF between E7A and ORF52 also shares similarity with select herpesvirus envelope glycoproteins. Furthermore, three ORFs in the 5′ end of the genome shared no homology to any viral or mammalian protein and are therefore labeled as unknown, abbreviated U4, U5, and U6.

Herpesvirus accessory ORFs are often viral homologues to proteins from the vertebrate host that they infect. Therefore, we sought to determine which mammalian species homologues were most closely related to each accessory ORF. A protein BLAST search followed by phylogenetic analysis was performed for each blue ORF in Fig. 6. The ORFs at the far left end of the genome, which include v-FLIP, v-OX2, DHFR, and v-Bcl-2, closely resembled both microbat and megabat proteins. A phylogenetic tree of v-OX2 and OX2 proteins from a variety of vertebrate species indicates that the viral homologue clusters with microbat sequences and that it is also near the megabat OX2 sequence (Fig. 6C). Since a subset of these ORFs share identity to bat gene sequences, this serves as additional evidence that the natural host for BGHV8 is a *Myotis* species bat and that the microbat cells from which the cell line was derived naturally harbored the virus prior to establishment of the cell line.

### mRNA profile of BGHV8 in MVI-it cell line.

**FIG 7:**
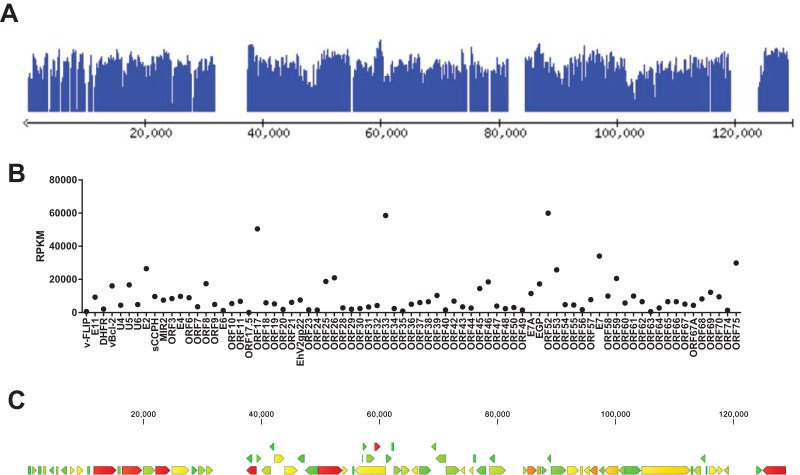
RNA-seq analysis from the MVI-it culture indicates that a majority of the BGHV8 coding sequences are transcribed. (A) Screen shot of RNA-seq reads mapped to each CDS of the BGHV8 genome. The data are presented in the Lightweight Genome Viewer (lwgv). The *y* axis is a log_10_ coverage scale, and the genomic position is displayed on the *x* axis. Interactive access to the coverage data across the BGHV8 genome is available at http://katahdin.mssm.edu/ravi/web/lwgv/lwgv.cgi?ann=nbghv_3.ann. (B) Reads per kilobase of transcript per million mapped reads (RPKM) for each CDS of BGHV8 are displayed. (C) A heat map imposed on the BGHV8 genome also illustrates the relative expression of each CDS. Green denotes the lowest RPKM values, yellow/orange indicates intermediate RPKM values, and red indicates the highest RPKM values. Raw reads and RPKM data are available through GEO (GSE76756).

We next utilized the RNA-seq data from the MVI-it cell line to profile the abundance of viral transcripts produced within the culture. Raw sequencing reads were mapped to the coding sequences (CDS) of the BGHV8 reference genome and are displayed in the Lightweight Genome Viewer (lwgv) (30). A representative screen shot (Fig. 7A) captures read coverage at each of the 77 CDS. The full genome map is available at http://katahdin.mssm.edu/ravi/web/lwgv/lwgv.cgi?ann=nbghv_3.ann. These coverage data, represented on a log_10_ scale, indicate expression of various genes and highlight that the majority of the genome is transcribed within the MVI-it cell line. An RNA-seq analysis in CLC Genomics Workbench which determined the reads per kilobase of transcript per million mapped reads (RPKM) further indicates that a majority of the genes are transcribed (Fig. 7B). The three most highly expressed genes were ORF17, ORF33, and ORF53 (all structural genes). A heat map imposed on the BGHV8 genome also illustrates the relative expression of each CDS (Fig. 7C). It is important to note that these data represent a single time point within a culture of cells where infection was not synchronized, but these data provide the basis for future studies to fully identify transcription start and stop sites and specific promoters.

## DISCUSSION

This study identifies what appears to be the first replication-competent bat gammaherpesvirus, an almost complete standard draft genome, and the first transcript map of a bat herpesvirus. As described in the ATCC product information sheet, the MVI-it cells were isolated from an “interscapular tumor; possibly basal cell” from an “adult female” bat. In addition, “the line was derived from a tumor found in a bat from Frio Cave, Texas. The tumor was in the skin, not attached to underlying muscle or bone.” The impetus to perform RNA-seq on the MVI cells was a desire to profile the innate immune response of bat cells to virus infection. The characterization of the innate immune transcriptome will be published elsewhere, but analysis of RNA-seq results led to the serendipitous identification of herpesvirus-like mRNA sequences. The identification of apparent full-length transcripts for viral structural proteins, including gB, glycoprotein H, major capsid protein, and tegument proteins, as well as proteins involved in virus genome replication, such as DNA polymerase, single-stranded DNA (ssDNA) binding protein, and thymidine kinase, points to ongoing virus replication in the cells. Further evidence for productive infection was obtained from electron microscopy images from the MVI-it cells which show the presence of what appear to be herpesvirus particles. The morphology and intracellular location are consistent with gammaherpesviruses. Although it is possible that cells in the culture harbor virus in a latent form and although we have not determined what percentage of cells contain virus genome, the fact that tissue culture supernatants from the MVI cells could transmit the infection to Vero cells proved the presence of ongoing productive replication as the cells were maintained in culture. Because it was from virus amplified on Vero cells that the full-length genome was determined, we can be certain that the genome sequence derives from an infectious virus. Since the genome sequence corresponds to the transcriptome data obtained in the bat cell line, it is clear that the virus growing on the Vero cells is the same virus that infects the bat cells.

To date, the best evidence for gammaherpesvirus infections of bats is based upon nucleotide sequences identified by deep sequencing or PCR-based approaches on bat-derived tissue samples (16–18, 31). These sequences correspond to only small portions of gammaherpesviruses, and isolation of infectious gammaherpesviruses has not been reported in the literature. Given that the virus was isolated from a cell line that was established many years earlier, one cannot be certain that the virus was present when the cells were first isolated. Therefore, it is difficult to be certain, based on the available data, that the virus is of bat origin. It could, for example, have been introduced during maintenance of the cells and have a nonbat source in nature. Nonetheless, several lines of evidence point to this being an actual bat gammaherpesvirus. First, the virus was isolated from cells of bat origin. Further, the cell line is of apparent tumor origin, consistent with the ability of gammaherpesviruses to transform infected cells (reviewed in references 32 and 33). Second, phylogenic analysis of gB indicates that the closest related sequence is from a sequence obtained from *Myotis* bat tissues. Additionally, phylogenetic analysis of selected ORFs with homology to mammalian host genes indicates a close relationship with microbat host gene products. Specifically, the most closely related gB sequence in GenBank to BGHV8 gB is a gB sequence obtained from *Myotis ricketti* bats, although the virus from which the sequence is derived has not been cultured*.* Finally, among 10 ORFs that do not share homology with EHV-2, seven were similar to proteins in other herpesviruses and therefore were assigned a name standard to other gammaherpesvirus accessory genes. These include v-FLIP, v-OX2, DHFR, v-Bcl-2, CCP, and MIR2 ORFs at the far 5′ end of the genome. We observed that several of these encode accessory proteins with homology to host factors and that the closest host homologue is a predicted bat protein. For example, v-FLIP has 36% identity to the CASP8 and FADD-like apoptosis regulator of *Myotis davidii*, v-OX2 has 68% identity to the OX2 membrane glycoprotein of *Myotis davidii*, v-DHFR has 68% identity to dihydrofolate reductase from *Myotis brandtii*, and MIR has 29% identity to the E3 ubiquitin-protein ligase MARCH2 of *Myotis brandtii*. A closer examination of v-OX2 reveals that the N terminus of the BGHV8 v-OX2 is quite similar to both KSHV v-OX2 and the *Myotis* OX2, with a more divergent C terminus. KSHV OX2, a secreted glycoprotein, activates myeloid lineage cells, resulting in proinflammatory cytokine production (34, 35). Although proof that this virus is found in nature in bats requires isolation of the virus from wild-caught animals, the findings outlined above provide support for a bat origin for the novel virus.

The structure of the BGHV8 genome is fairly typical of a gammaherpesvirus. Nonetheless, there is little nucleotide homology with publicly available gammaherpesvirus sequences. Therefore, genome annotation was based upon homology to other gammaherpesvirus proteins. Upon initial ORF analysis of BGHV8, we did find that a majority of ORFs are relatively closely related to EHV-2 (identity, ~75% or lower) using a specialized gammaherpesvirus database in VIGOR. A majority of ORFs share some protein identity with EHV-2 (36) and comprise both structural proteins and proteins involved in viral transcription and replication. Moreover, BGHV8 has a genomic organization consistent with a gammaherpesvirus genome, including the ORF06, ORF07, ORF08, and ORF09 cluster at the left side of the genome and the ORF67A, ORF68, and ORF69 cluster at the right side of the genome (37). In addition, the presence of a putative miRNA cluster in a noncoding region at the right end of the genome (between bases 121326 and 121820) strikingly corresponds to a region within KSHV between bases 119000 and 122000 that carries a cluster of 10 miRNAs that promote cellular transformation (26, 38).

Given the association of bat viruses with human disease, the capacity of BGHV8 to infect human cells is of interest. Our *in vitro* data indicate that BGHV8 can be propagated in Vero cells and can also productively infect additional cell lines, including some human cell lines, and confirmed viral replication between 1 and 5 days postinfection in several cell lines. Due to its ability to infect human cell lines, we strongly recommend biosafety level 2 precautions when handling BGHV8. BGHV8 clearly clusters with gammaherpesviruses (Fig. 5 and 6). BGHV8 also has a classic ORF49/ORF50 cassette and a putative LANA gene (Fig. 4), suggesting that it has the ability to establish latency, a focus of future work.

The majority of all emerging infectious diseases (EIDs) arise from zoonotic pathogens (1), and bats are of significant interest as potential reservoir hosts for zoonotic viruses, including filoviruses, henipaviruses, coronaviruses, lyssaviruses, herpesviruses, and others (15, 39–44). Bats belong to the order Chiroptera, which is divided into two suborders, Megachiroptera (megabats), containing a single family, Pteropodidae, with 42 genera, and Microchiroptera (microbats), containing 16 families with 135 genera. Bats evolved roughly 50 million years ago and have diverged into 925 known species comprising 20% of all known mammal species. Several hypotheses have been put forth as to why bats are frequent reservoirs for emerging viruses, including their habitation in colonies; their existence as the only flying mammal, which might allow viral dissemination across a large geographic distance; and their long life spans relative to their body size (29, 45). Testing these hypotheses will be facilitated by the availability of viruses that naturally infect bats. The cell line derived from *Myotis velifer incautus* is present in Texas and surrounding regions, consistent with the isolation of the tumor from Frio Cave in Texas (46). *Myotis lucifugus*, a related species, is a predominant bat species across the majority of the United States and parts of Canada. To our knowledge, viruses isolated from *M. velifer incautus* bats are not described. Given the similarity to *M. lucifugus*, it is likely that these two species would harbor similar pathogens. The fact that this cell line is derived from a tumor is interesting, since tumors in bats are presumed to be rare and DNA repair proteins are highly expressed (29). While our work validates the qPCR assay, future efforts to develop a serological assay to detect the virus’s prevalence are desired.

Replication-competent BGHV8 and its annotated genome provide a valuable tool to further understand bat-virus interactions. Many of the ORFs described above are likely to modulate apoptosis (e.g., v-FLIP and vBcl-2), cell signaling (the BGHV G-protein-coupled receptors), and cytokine production (e.g., v-OX2). Moreover, inhibition of the cGAS DNA sensing pathway has been linked to KSHV ORF52 (47), and a corresponding ORF52 is present in BGHV8 (Fig. 4). In the future, defining how selected BGHV8 ORFs modulate innate and adaptive immune signaling pathways in bats should provide a powerful and unique tool to further understand bat biology.

## MATERIALS AND METHODS

### RNA sequencing and analysis of the MVI-it cell line.

Cell line CRL-6012 was obtained from the American Type Culture Collection (ATCC) and passaged until cells reached a confluent monolayer in a 75-centimeter flask. Cells were maintained in Dulbecco’s modified Eagle’s medium (DMEM) supplemented with 10% fetal bovine serum. RNA was isolated with Trizol (Invitrogen), and mRNA was subjected to next-generation sequencing on the Genome Analyzer II (42-bp single-read data). *De novo* assembly of raw read data generated 5,818 contigs, labeled as BatA1 to BatA5818. Nucleotide sequences from each contig were translated into protein space, and protein sequences were subjected to BLAST search against both the human and nonredundant protein databases, uncovering contigs with homology to herpesvirus protein sequences. For RNA-seq analysis, sequencing reads were mapped against each BGHV8 coding sequence (CDS) and in CLC Genomics Workbench (version 8.5) using the CLC RNA-seq tool. Total reads and the RPKM for each CDS were determined.

### Isolation and propagation of BGHV8 virions.

Strict biosafety level 2 precautions were followed for all studies involving live virus. Supernatant obtained from a confluent 75-centimeter flask of MVI-it cells was harvested and clarified by centrifugation at 3,500 rpm for 20 min. Supernatant was transferred to uninfected Vero cells (ATCC CCL-81) seeded in 6-well plates. At either 18 or 42 h after supernatant transfer, supernatant was harvested and stored and cells were fixed in 2% paraformaldehyde (PFA). Phase-contrast images captured evidence of cytopathic effect, and Hoechst staining (Thermo Fisher) captured evidence of syncytium formation. To generate working stocks of BGHV8, MVI-it supernatant was passaged onto confluent flasks of Vero cells, and supernatant was harvested once complete CPE was apparent. Viral titers of BGHV8 were calculated by either plaque assay or 50% tissue culture infectious dose (TCID_50_) assay on Vero cells. For plaque assay, serial dilutions of clarified supernatant were used to infect fresh Vero cells in 6-well plates for 2 h. Infection medium was removed, and cells were overlaid with Avicel-591. Briefly, a 2.4% Avicel solution was mixed 1:1 with 2× DMEM and 4 ml of this mixture was added to each well of a 6-well plate. Three to 4 days postinfection, the Avicel overlay was removed, and cells were washed with phosphate-buffered saline (PBS), fixed with 4% PFA, and stained with crystal violet to visualize plaques. For TCID_50_ studies, Vero cells were seeded in 96-well plates. Serial 10-fold dilutions of BGHV8 stocks were added in replicates of 7 adjacent columns. When complete CPE was visible at lower dilutions, cells were fixed with PFA and stained with crystal violet.

### BGHV8 infection of cell lines.

Both TCID_50_ and plaque assays were used to calculate viral stock titers. BGHV8 stocks were used to infect target cells at a multiplicity of infection (MOI) of approximately 0.5 PFU/cell. Cell lines tested included mouse embryonic fibroblasts, Hepa1.6, Huh7, MRC, A549, HeLa, and Vero. Both uninfected cells and cells at days 1, 3, and 5 postinfection were collected for total RNA. Corresponding supernatant from each sample was also harvested, and DNA from supernatants was isolated (QIAamp blood kit). From total RNA, cDNA was generated using oligo(dT) primers (Superscript III; Invitrogen). Quantitative PCR primers designed against a BGHV8 capsid mRNA sequence identified in the initial RNA-seq study were used to measure BGHV8 RNA in cells. The forward primer sequence is 5′ ACACCAACAGAGACCCCGCC 3′, and the reverse primer sequence is 5′ AGCACGGTCTGGCTTACTTTGCG 3′. To measure particle release from infected cell supernatants, quantitative PCR from isolated DNA was performed with the same primer set.

### Transmission electron microscopy.

For ultrastructural analysis in ultrathin sections, *Myotis* bat cells were fixed for at least 1 h in a mixture of 2.5% formaldehyde prepared from paraformaldehyde powder and 0.1% glutaraldehyde in 0.05 M cacodylate buffer (pH 7.3) to which 0.03% picric acid and 0.03% CaCl_2_ were added. The monolayers were washed in 0.1 M cacodylate buffer, and cells were scraped off and processed further as a pellet. The pellets were postfixed in 1% OsO_4_ in 0.1 M cacodylate buffer (pH 7.3) for 1 h, washed with distilled water, and *en bloc* stained with 2% aqueous uranyl acetate for 20 min at 60°C. The pellets were dehydrated in ethanol, processed through propylene oxide, and embedded in Poly/Bed 812 (Polysciences, Warrington, PA). Ultrathin sections were cut on a Leica EM UC7 ultramicrotome (Leica Microsystems, Buffalo Grove, IL), stained with lead citrate, and examined in a Philips 201 transmission electron microscope at 60 kV.

### BGHV8 genome construction and annotation.

Extracted DNA from BGHV8-infected Vero cells was sheared (Covaris), and a library was generated for sequencing by Illumina HiSeq2500 to generate 100-bp reads. A second library from viral genomic DNA was generated by sequence-independent single primer amplification (SISPA) as previously described (23) and was sequenced on the Illumina MiSeq platform to generate trimmed sequencing reads approximately 270 bp in length. *De novo* assembly in CLC Genomics Workbench resulted in several contigs. Using the CLC genome finishing tool, longer MiSeq reads were used to join the contigs generated from the HiSeq data into a single sequence of approximately 130 kb. To annotate the assembled genome, we developed gammaherpesvirus-specific annotation software called Viral Genome ORF Reader (VIGOR) (24), with very relaxed parameters on the percent similarity cutoff to accommodate the variation in herpesvirus genomes. VIGOR was used to predict genes, perform alignments, ensure the fidelity of open reading frames, and detect any potential sequencing or assembly errors. The annotation was manually verified and edited as required, according to the gene and protein naming convention of the *Equid herpesvirus 2* (EHV-2) reference genome (NC_001650.2). We analyzed the assembled BGHV8 sequence in a tandem repeat finder program (http://tandem.bu.edu/trf/trf.html) which was previously used to identify repeat regions in a mouse gammaherpesvirus genome (25).

### BGHV8 phylogenic analyses.

Phylogenic analyses were performed on the BGHV8 glycoprotein B (gB) and v-OX2 protein sequences. For gB, a protein BLAST search using gB was performed, results were used to create alignments, and sequences were aligned and trimmed prior to generation of a tree. An outgroup, human herpesvirus 1, was used to generate a phylogenetic tree using the LG substitution model and 100 bootstraps and root it on the outgroup. For v-OX2, a protein BLAST search against all viral sequences was performed, resulting herpesvirus sequences were aligned and trimmed, and a tree was constructed using methods similar to those described for gB. A v-OX2 protein BLAST search was also performed against the mammalian protein database. Results were aligned and trimmed, and a tree was constructed using methods described above.

### Accession numbers.

The annotated genome can be accessed through BioProject ID PRJNA 295864 and GenBank accession number KU220026. The RNA-seq reads mapped to the annotated genome are accessed through BioProject ID PRJNA 295864, and the GEO accession number is GSE76756.
